# The alliance of genome resources: transforming comparative genomics

**DOI:** 10.1007/s00335-023-10015-2

**Published:** 2023-09-04

**Authors:** Carol J. Bult, Paul W. Sternberg

**Affiliations:** 1 The Jackson Laboratory, Bar Harbor, ME USA; 2 California Institute of Technology, Pasadena, CA USA

## Abstract

Comparing genomic and biological characteristics across multiple species is essential to using model systems to investigate the molecular and cellular mechanisms underlying human biology and disease and to translate mechanistic insights from studies in model organisms for clinical applications. Building a scalable knowledge commons platform that supports cross-species comparison of rich, expertly curated knowledge regarding gene function, phenotype, and disease associations available for model organisms and humans is the primary mission of the Alliance of Genome Resources (the Alliance). The Alliance is a consortium of seven model organism knowledgebases (mouse, rat, yeast, nematode, zebrafish, frog, fruit fly) and the Gene Ontology resource. The Alliance uses a common set of gene ortholog assertions as the basis for comparing biological annotations across the organisms represented in the Alliance. The major types of knowledge associated with genes that are represented in the Alliance database currently include gene function, phenotypic alleles and variants, human disease associations, pathways, gene expression, and both protein–protein and genetic interactions. The Alliance has enhanced the ability of researchers to easily compare biological annotations for common data types across model organisms and human through the implementation of shared programmatic access mechanisms, data-specific web pages with a unified “look and feel”, and interactive user interfaces specifically designed to support comparative biology. The modular infrastructure developed by the Alliance allows the resource to serve as an extensible “knowledge commons” capable of expanding to accommodate additional model organisms.

## Introduction

**Fig. 1:**
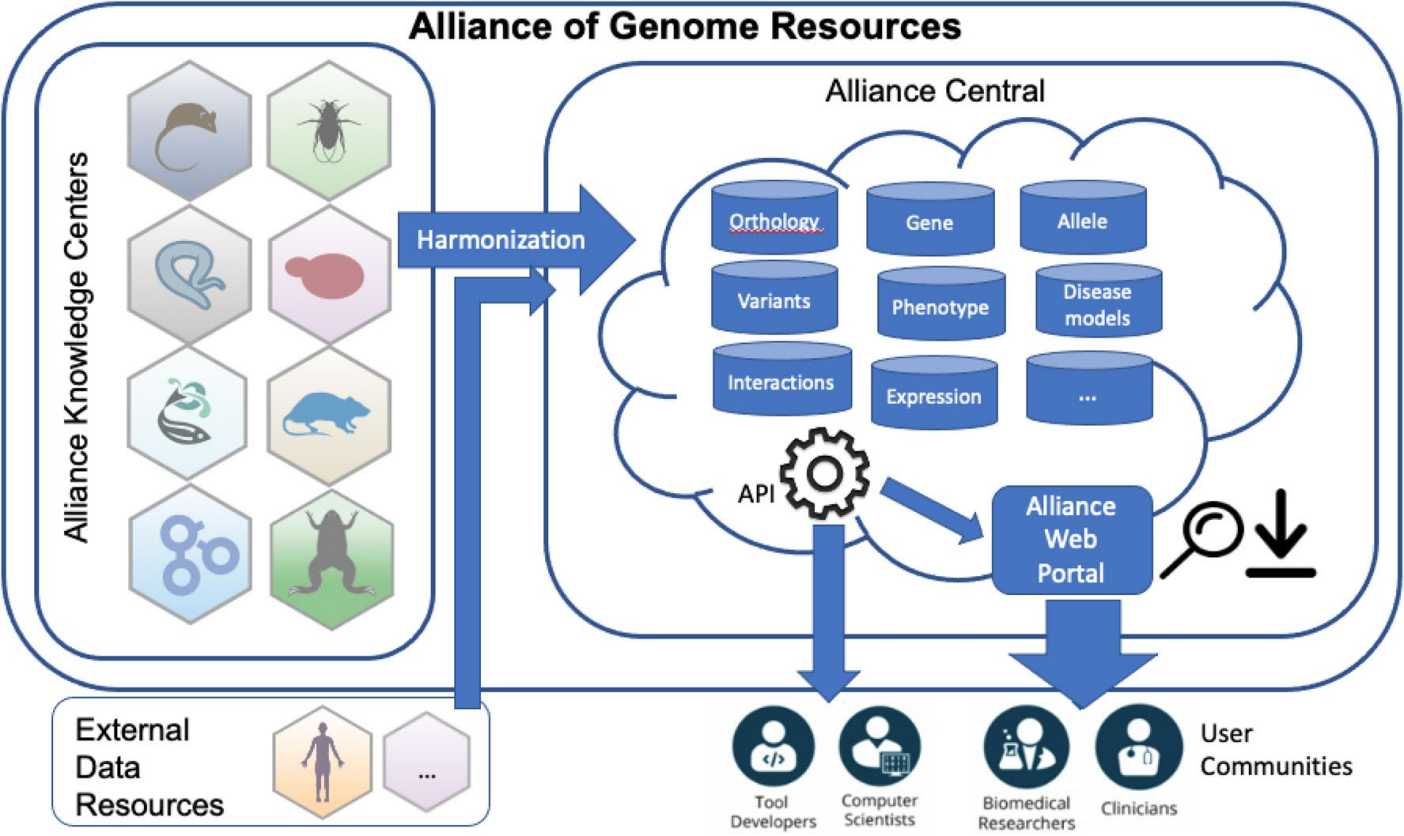
A graphical representation of the organizational units that comprise the Alliance of Genome Resources consortium. Knowledge Centers (aka, model organism databases or MODs) are organism-specific resources for expert curation of knowledge about a model organism’s genome. Data from external resources are integrated with genome annotations provided by the Knowledge Centers. Alliance Central is the platform for delivery of harmonized biological annotations to a diverse user community via the Alliance web portal and Application Programming Interfaces (APIs)

The Alliance of Genome Resources (the Alliance) is a consortium of seven model organism knowledgebases and the Gene Ontology resource. The mission of the Alliance is to support comparative genomics as a means to investigate the genetic and genomic basis of human biology, health, and disease. The Alliance seeks to serve a diverse community of biomedical researchers including basic scientists, clinicians, and data scientists. To promote sustainability of core community data resources, the Alliance has implemented and maintains an extensible “knowledge commons platform” for comparative genomics using modular infrastructure components that can be used by a wide range of multiple model organism genome knowledgebases (Alliance of Genome Resources 2019, 2020; Howe et al. 2018). The history of how Model Organism Databases and the Gene Ontology Consortium united to form the Alliance of Genome Resources has been published previously (Alliance of Genome Resources 2019, 2022). In 2023, the Alliance was recognized as a Core Global Biodata Resource by the Global Biodata Coalition (Anderson et al. 2017) (Fig. 1).

The Alliance of Genome Resources is organized as two interdependent units: Alliance Central and the Alliance Knowledge Centers (Alliance of Genome Resources 2019) (Fig. 1). *Alliance Central* is responsible for developing and maintaining the software for data access and for the coordination of concept modeling and data harmonization activities across the Knowledge Centers. The ultimate goal of Alliance Central is to reduce redundancy in systems administration and software development for model organism data resources and to deploy a unified ‘look and feel’ for access to and display of data types and annotations in common across diverse model organisms. Model organism-specific knowledgebases serve as *Alliance Knowledge Centers.* Knowledge Centers are responsible for expert curation of data and for submission of data to Alliance Central using standardized data formats and annotation standards. Knowledge Centers also are responsible for organism-specific user support activities and for providing access to data types not yet supported by Alliance Central. The founding Alliance Knowledge Centers are Saccharomyces Genome Database (Engel et al. 2022), WormBase (Davis et al. 2022), FlyBase (Gramates et al. 2022), Mouse Genome Informatics (Ringwald et al. 2022), the Zebrafish Information Network (Bradford et al. 2023), Rat Genome Database (Vedi et al. 2023), and the Gene Ontology Consortium (Gene Ontology et al. 2023). The newest member, Xenbase (Fisher et al. 2023), joined the Alliance consortium in 2022. Annotations for human genes are acquired from numerous resources including the Alliance Knowledge Centers, NCBI’s dbSNP (Smigielski et al. 2000), the Human Gene Nomenclature Committee (Yates et al. 2021), Disease Ontology (Schriml et al. 2022), Human Phenotype Ontology (Kohler et al. 2021), Orphanet (Rath et al. 2012), OMIM (Hamosh et al. 2021), BioGrid (Oughtred et al. 2021), and Reactome (Gillespie et al. 2022).

**Fig. 2:**
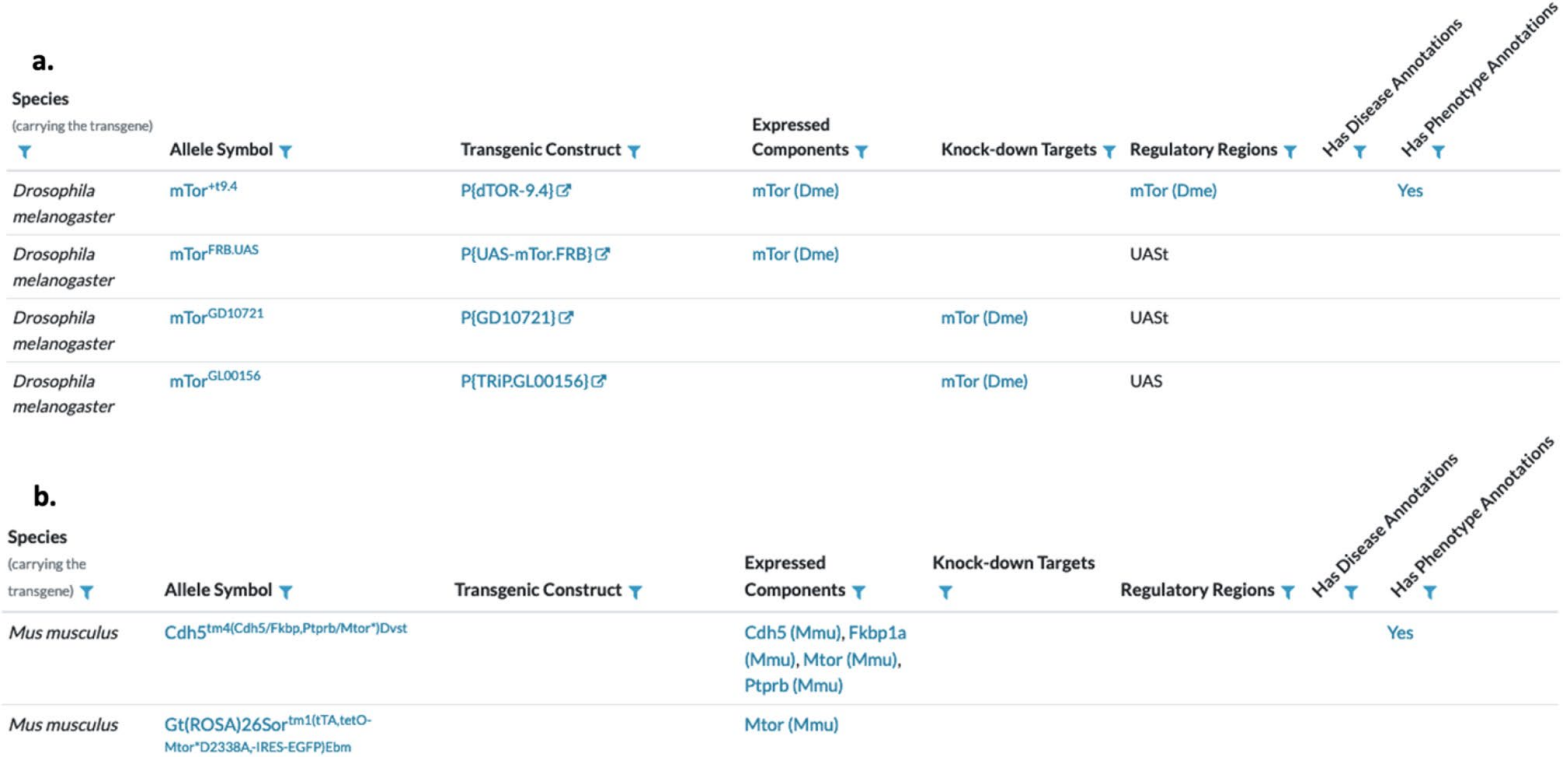
Example of the harmonized representation of transgenic alleles at the Alliance of Genome Resources. **a** Transgenic alleles of the mTor gene in *Drosophila*. **b** Transgenic alleles of the *Mtor* gene in the laboratory mouse. In the Alliance data model, transgenic alleles are represented by various components that can be populated with information in a species-specific manner which allows for representational consistency and completeness across all model organisms. Allele symbols conform to species-specific nomenclature standards. Transgenic constructs represent the transgene construct independent of the host organism following species-specific guidelines. Expressed components represent the genetic elements expressed by the construct which may be full or partial genes and may include both protein-coding and non protein-coding genes. Knock-down targets are elements in the transgene designed to interfere with the expression of another gene. The regulatory regions component includes genetic elements driving the expression of entities in the construct (e.g., upstream activation sequence, human cytomegalovirus, etc.)

Although the model organism-centric knowledgebases that comprise the Alliance all contain similar data types (e.g., gene function, gene expression, genetic variation, phenotype, and human disease associations), the resources differ in how these data types are modeled and displayed to end users. These differences present significant challenges to the development of common schemas and uniform user interfaces for data types across different organisms. To address these challenges, a major activity within the Alliance is the harmonization of biological concepts which can be represented in a common schema. For example, all of the model organisms currently in the Alliance consortium have a concept of a transgene. For some model organisms, a transgene is represented as a random insertion of a construct but does not include gene trap alleles. For other organisms, gene traps are included in the representation of transgenes. For yet other model organisms, transgenes are represented as the random insertion of any foreign DNA into the genome, including the construct. To implement a common data model and unified display for transgenes, a harmonized data model was developed in which transgenes are represented by two separate concepts—the transgene construct and the transgene allele—and the specific relationships between the concepts. In the harmonized Alliance model, a transgene construct is defined as the DNA used to create a transgenic allele. The transgene construct has explicit relationships to genes, gene segments, and to the transgenic alleles created using the construct. The transgenic allele represents a construct in the context of a genome. Transgenic alleles have relationships to constructs and genomes. Because the transgene data type is harmonized, data from all of the model organism-specific Knowledge Centers can be represented in a uniform manner on the Alliance web portal (Fig. 2).

Biological annotations obtained from data-specific resources that are not members of the Alliance consortium are also incorporated into Alliance Central. For example, the Biological General Repository for Interaction Datasets (BioGrid) (Oughtred et al. 2021) and the International Molecular Exchange consortium (IMex) (Porras et al. 2022) are primary sources of molecular and genetic interaction data. Reactome (Gillespie et al. 2022) is leveraged as one source of pathway and reaction data. The Alliance Central practice of leveraging existing community resources also extends to software for data analysis and visualization. Externally developed tools such as Intermine (Smith et al. 2012), JBrowse (Buels et al. 2016), Apollo (Dunn et al. 2019), SequenceServer (Priyam et al. 2019), and the Reactome pathway viewer (Gillespie et al. 2022) are key components of the knowledge commons platform providing useful functionality for the Alliance user community and allowing software development efforts within Alliance Central to be focused on tools and interfaces for comparative biology and genomics that provide added value to the biomedical research community. A number of the software components used by the Alliance (e.g., Apollo, JBrowse, Intermine) were developed under the auspices of the Generic Model Organism Database project (http://gmod.org/wiki/Main_Page).

The Alliance resource has a unique and complementary role relative to other informatics resources that support comparative biology such as NCBI’s new Comparative Genomics Resource (CGR; https://www.ncbi.nlm.nih.gov/comparative-genomics-resource/). Whereas the CGR is focused on developing analysis tools and resources for sequence-based genome comparisons across a large number of species, the Alliance focuses on standardized annotations, harmonized biological concepts, and comparison of biological knowledge. The CGR supports comparative sequence analysis for all eukaryotes whereas the Alliance is primarily focused on model organisms used widely in biomedical research. The CGR resource integrates the standardized gene summaries from the Alliance and follows nomenclature and ontology standards developed and maintained by Alliance members. For sequence analysis, the Alliance leverages sequence-based analysis tools developed and maintained by the CGR such as BLAST.

The approach to data management for the Alliance and its members aligns with modern FAIR principles (Findability, Accessibility, Interoperability, and Reusability) (Wilkinson et al. 2016) which are designed to ensure that data are structured to be machine accessible with minimal or no human intervention. Examples of how the Alliance conforms to FAIR principles includes the use of unique, persistent identifiers for data entities and meta-data, the use of well-recognized and accepted community standard bio-ontologies and vocabularies for knowledge representation, clear data use licensing guidelines, and the availability of open and freely available application programming interfaces (APIs) for data retrieval.

## The Alliance of Genome Resources web portal

**Fig. 3:**
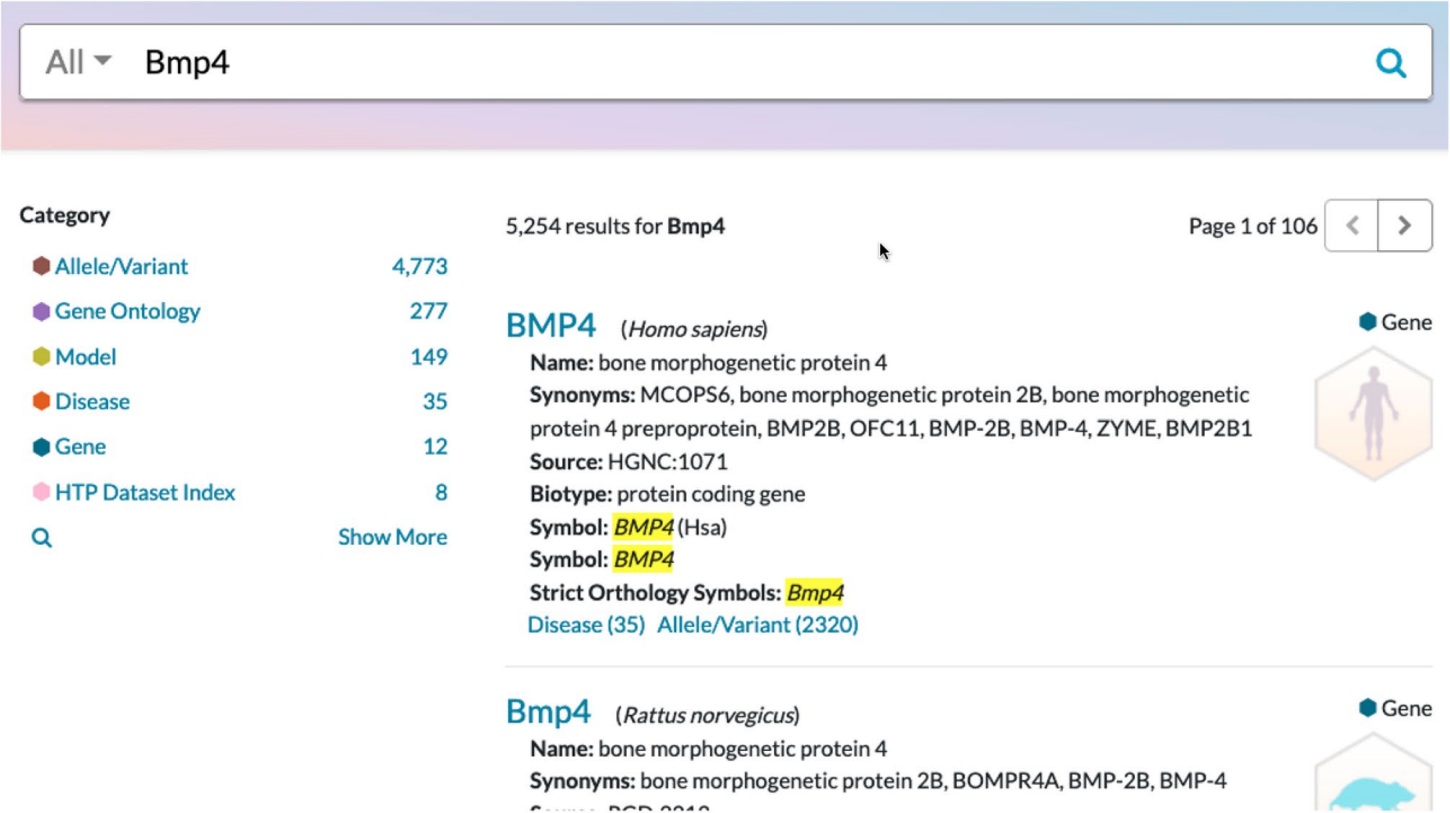
Partial screenshot of the Alliance web portal results page showing the search results for the *BMP4* gene. The six major categories of data with counts of database records are provided on the left navigation panel. Users can refine their searches by selecting a category of interest which leads to the display of new facet categories and record counts. Alternatively, users can scroll through the search results and select a gene of interest to navigate to the relevant detail page in the web portal

The Alliance web portal (www.alliancegenome.org) provides a single point of access to the expertly curated and harmonized annotations from diverse model organisms and humans. The portal supports keyword searching within six categories: Gene, Allele/Variant, Disease Models, Gene Ontology annotation, Disease, and High Throughput Data (HTP) (Fig. 3). Results of keyword searches are displayed as faceted counts for the six categories. The counts are updated as search parameters are refined by the user. The Alliance database content summary as of the most recent release of the portal (v. 5.4.0) is provided in Table 1.

**Table 1:** Content summary for the alliance of genome resources web portal (v. 5.4.0)

Data type	Count
Genes	352,073
Alleles/variants	401,287,981
Disease models	142,147
Functional annotations	43,095
Disease ontology (DO) terms Annotations using DO terms	11,237 351,137
High throughput datasets	10,753

Data at the portal is available currently for human, mouse, rat, zebrafish, frog, nematode, yeast, and fruit fly

**Fig. 4:**
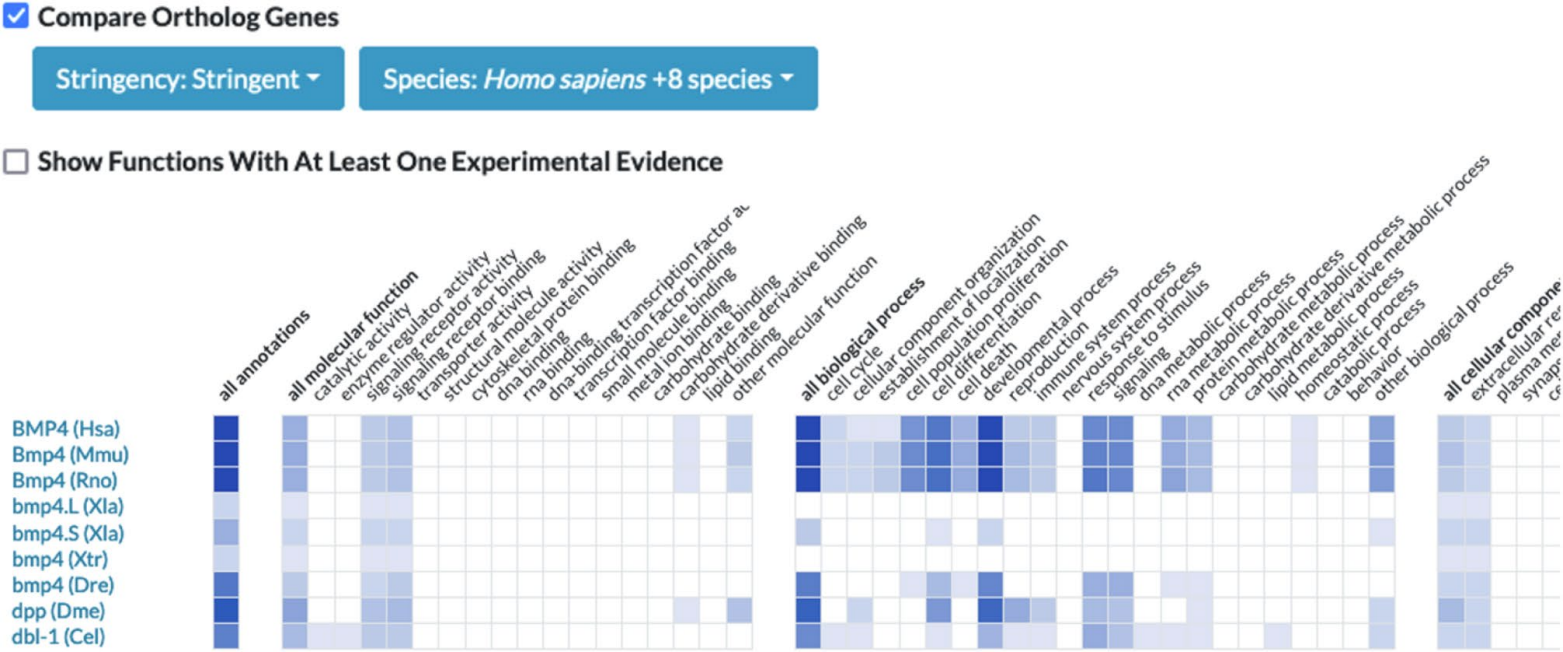
Screenshot showing the interactive annotation summary ribbon for functional annotations of orthologs of the human *BMP4* gene. Each row in the ribbon is a different species. Each column is a grouping of terms from the molecular function, biological process, and cellular component categories of the Gene Ontology. The shading in the cells reflects the number of annotations to terms represented by the column. The darker the shading, the more annotations. Selecting a cell in the ribbon results in a table that shows the specific ontology terms and evidence used to make the annotation. Users can use the orthology settings to control which organisms are shown in the graphic

Search results are displayed with a consistent look and feel and layout of data for all organisms represented in the Alliance. To facilitate the comparison of biological knowledge across multiple species, annotations for orthology, function, phenotype, and disease are displayed in the portal use an interactive comparative annotation ribbon (Fig. 4). The ribbon display allows users to quickly assess the degree to which annotations are similar across multiple species. The cells in the ribbon are linked to tabular summaries with details about the relevant ontology terms and sources of evidence for the annotations.

In addition to support for keyword searches, the Alliance web portal provides users with downloadable files of gene description summaries and annotations in commonly used data formats (e.g., JSON, tab-separated, GFF, etc.). Downloads are currently available for disease annotations, gene expression, molecular and genetic interactions, orthology, alleles, and short gene descriptions. Sequence variants that are associated with documented phenotypic consequences are available as files in Variant Call Format (VCF) format. The downloadable data files are updated regularly. The file headers display the Alliance database version and the date the file was generated. Programmatic access to annotations in the Alliance is provided through an OpenAPI Specification (OAS). The schemas for the API-accessible data classes are available in a browsable format on the website.

Users looking to search for more than one gene at a time or in managing lists of genes can use AllianceMine. AllianceMine uses the InterMine data warehouse system (Smith et al. 2012). Although AllianceMine can be used without creating an account, having an account allows users to save gene lists and the outputs of gene list operations (i.e., intersection, combine, difference, subtraction).

Schedules for public data releases at the Alliance Knowledge Centers range from daily to monthly. Data submitted from Alliance Knowledge Centers and other external data sources are refreshed monthly at the Alliance web portal. These monthly data releases are occasionally suspended when major software changes to the Alliance infrastructure are being implemented. Release notes are accessed from the News menu in the header that document changes to data release frequencies, user interfaces, portal functionality, and any known issues with Alliance resources (https://www.alliancegenome.org/release-notes).

## Orthology

Gene orthology is fundamental to comparative genomics. The Alliance uses a common set of orthologs as the foundation for comparing functional, phenotype, and disease annotations across model organisms and humans. Alliance orthology assertions are based on outputs from algorithms/methods that have been benchmarked by the Quest for Orthologs Consortium (Nevers et al. 2022) and integrated using the DRSC Integrative Ortholog Prediction Tool (DIOPT) (Hu et al. 2011). These ortholog assertions are subsequently supplemented with manually curated ortholog inferences from the Human Gene Nomenclature Committee (for human and mouse genes) (Yates et al. 2021), Xenbase (for frog genes), and ZFIN (for zebrafish genes). Manual curation is particularly useful for ensuring accuracy and completeness of orthology representation for species such as *Xenopus* and *Danio* where there has been extensive genome duplication.

## Gene detail pages

Organism-specific gene detail pages in the Alliance web portal are the primary ‘hub’ of functional and biological annotations. All gene pages include a summary section with a short description of what the gene does and its association with phenotypes and/or human disease. The gene function descriptions are generated automatically by an algorithm that leverages expertly curated structured ontology term annotations associated with genes (Kishore et al. 2020). In addition to a gene summary, the standard sections of gene pages include Orthology, Function, Pathways, Phenotypes, Disease Associations, Models, Alleles and Variants, Transgenic Alleles, Sequence Feature Viewer, Gene Expression, Molecular Interactions, and Genetic Interactions. The two primary means for displaying annotations in these categories are a table view and an annotation ribbon display. Brief descriptions for each section of the gene detail pages and examples of the display paradigms are provided below. A list of the specific ontologies used at the Alliance along with licensing information is provided in Table 2 and on the Privacy, Warranty, and Licensing page (https://www.alliancegenome.org/privacy-warranty-licensing) at the Alliance web portal.

**Table 2:** Ontologies used within the alliance of genome resources to annotate function, phenotype, and disease properties associated with genome features, gene products, genetically defined populations, and other biological entities

		Alliance knowledge center						
Ontology	Abbreviation	ZFIN	MGD	SGD	WormBase	FlyBase	RGD	Xenbase
Ascomycete phenotype ontology	APO			✓				
Biological spatial ontology	BSPO	✓						
*C. elegans* (nematode) life stage	WBls				✓			
*C. elegans* anatomy	WBbt				✓			
*C. elegans* phenotype	WBPhenotype				✓			
Cell ontology	CL		✓				✓	
Chemical entities of biological interest	ChEBI	✓	✓	✓	✓		✓	
Clinical measurement ontology	CMO						✓	
Drosophila development	FBdv					✓		
Drosophila gross anatomy	FBdt					✓		
Drosophila phenotype ontology	DPO					✓		
Embrace data and methods	EDAM			✓				
Evidence and conclusion ontology	ECO	✓	✓	✓			✓	
Experimental condition ontology	XCO						✓	
FlyBase controlled vocabulary	FBcv					✓		
Gene ontology	GO	✓	✓	✓	✓	✓	✓	✓
Human disease ontology	DOID	✓	✓	✓		✓	✓	✓
Human phenotype ontology	HP		✓				✓	
Mammalian phenotype ontology	MP		✓				✓	
Measurement method ontology	MMO						✓	
Molecular interactions	MI			✓		✓	✓	
Mouse adult gross anatomy	MA		✓				✓	
Mouse developmental stage ontology	Mmusdv		✓					
Mouse gross anatomy and development, timed	EMAPA		✓					
Mouse pathology	MPATH	✓	✓					
Ontology for biomedical investigations	OBI			✓				
Pathway ontology	PW						✓	
Phenotype and trait ontology	PATO	✓	✓		✓			
Protein modification	MOD			✓				
Protein ontology	PRO		✓					
Rat Strain ontology	RS						✓	
Relations ontology	RO	✓	✓	✓	✓			
Sequence types and features	SO	✓	✓	✓		✓	✓	
Uberon	Uberon		✓				✓	
Vertebrate trait ontology	VT		✓					
Xenopus anatomy ontology	XAO							✓
Xenbase experimental data ontology	XBED							✓
Xenopus phenotype ontology	XPO							✓
Xenopus small molecule ontology	XSMO							✓
Zebrafish anatomy	ZFA	✓						
Zebrafish developmental stages	ZFS	✓						
Zebrafish experimental conditions ontology	ZECO	✓						

### Orthology

**Fig. 5:**
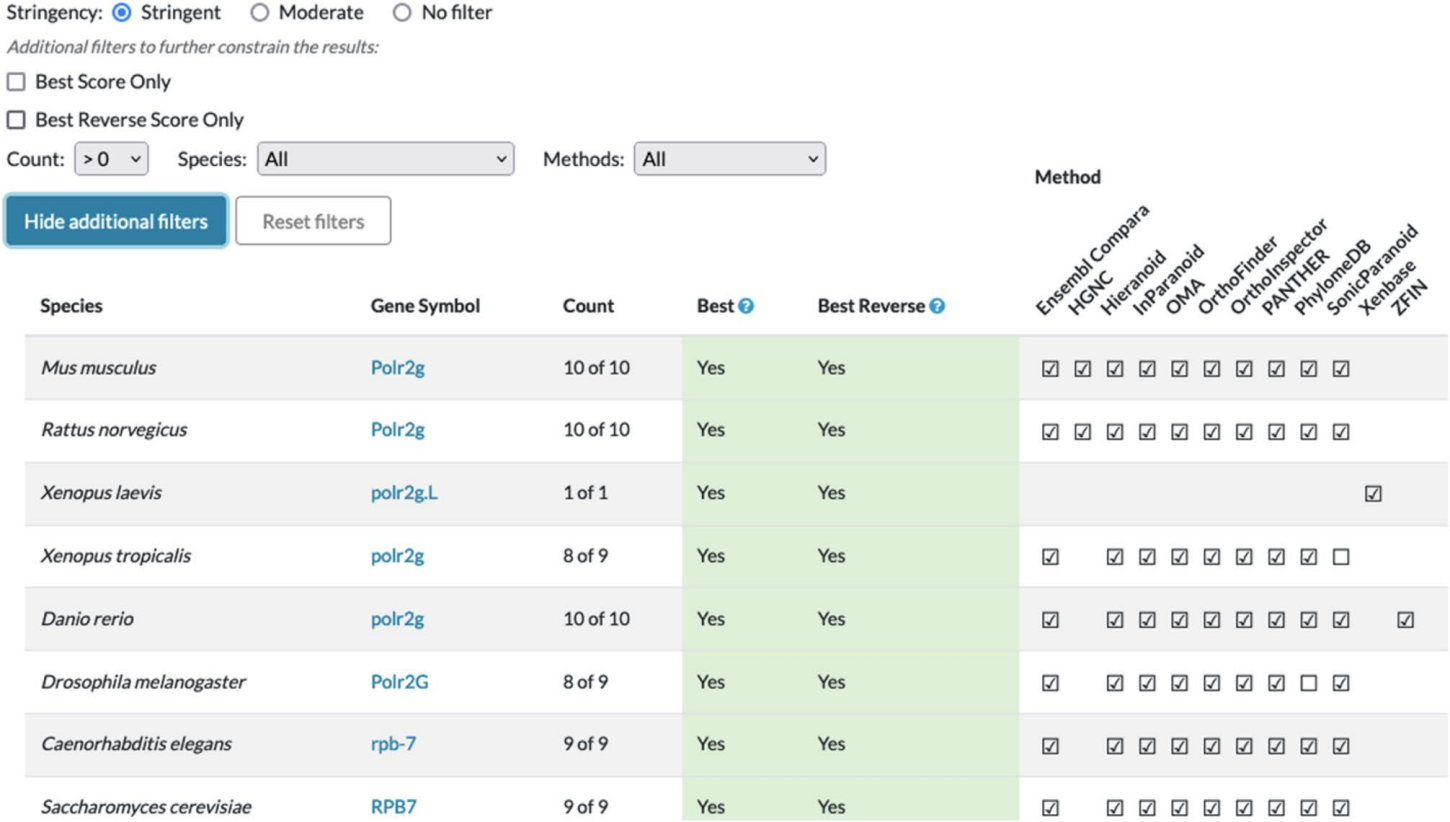
Screenshot of the orthology summary for the human *POLR2G* gene. By default, only orthologs with high stringency (as determined by DIOPT score) are displayed. Users can adjust the settings for stringency, species displayed, and orthology call methods used. Alliance orthologs are also available as a downloadable file and via the Alliance API

The default display of orthologs reflects the output of the most stringent criteria based on DIOPT score; however, options are provided for researchers to select less stringent criteria and/or orthologs predicted by a user-selected subset of the available inference methods (Fig. 5). The Alliance orthologs are available as a downloadable file and can also be accessed programmatically via the Alliance Central API service.

### Functional annotations (Gene Ontology)

Annotations to terms for Biological Process, Molecular Function, and Cellular Component from the Gene Ontology are summarized for high level GO categories using the Alliance annotation ribbon display paradigm (Fig. 4). Each cell in the ribbon is shaded if there is an annotation for a term in the category. The deeper the color of the shading, the more annotations are associated with the terms in the category. Selecting a cell generates a table that lists all of the annotation terms with evidence codes and sources for the annotation(s). By expanding the display to include additional organisms, the functional annotations for orthologs are displayed as additional rows.

### Pathways

**Fig. 6:**
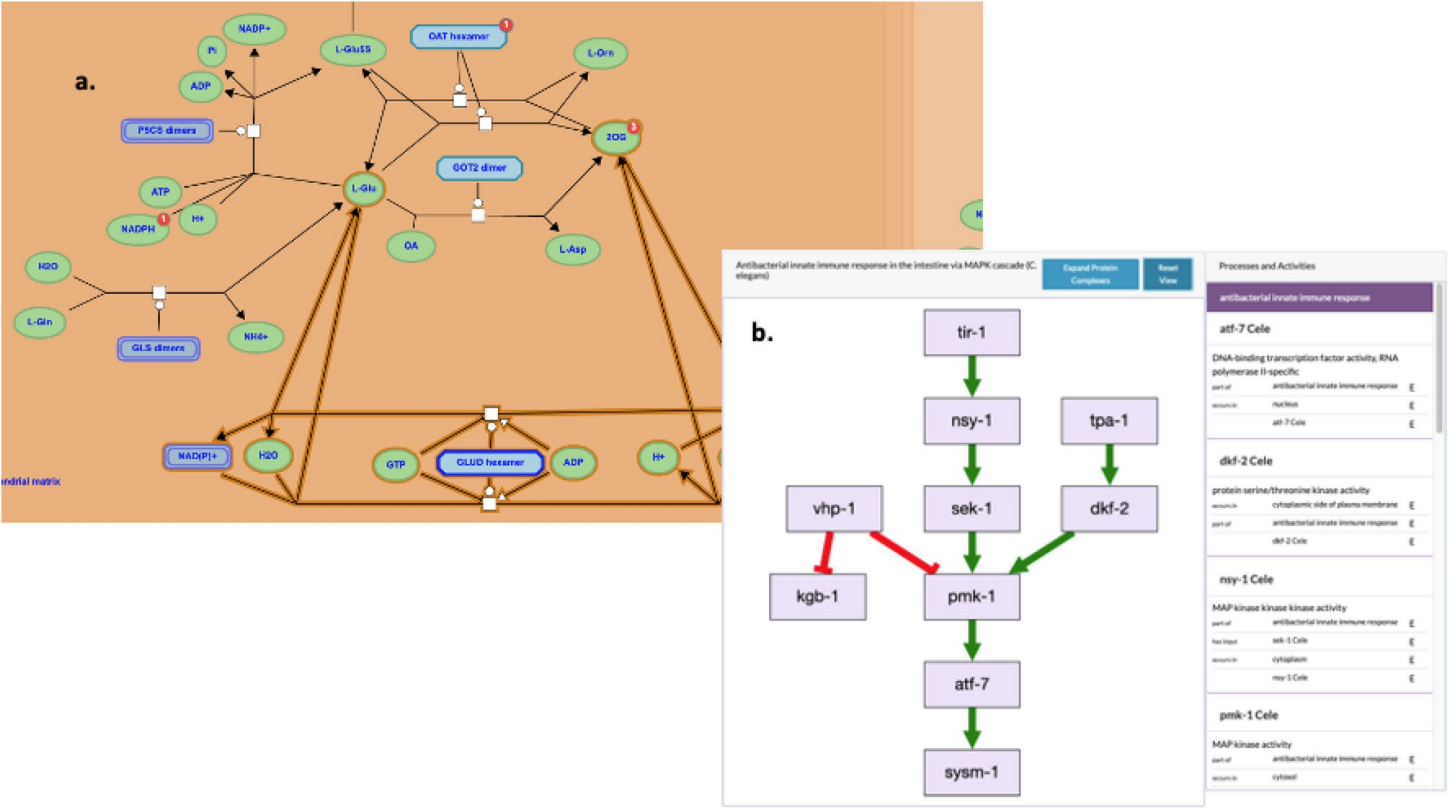
Screenshots showing pathway representation in the Alliance web portal. **a** Pathway summary display for glutamate and glutamine metabolism for mouse *Glud1* from Reactome. The display is integrated into the gene detail pages and links out to the Reactome resource. **b** Gene Ontology causal activity model (GO-CAM) representation of the antibacterial innate immune response in the intestine via MAPK cascade biological process associated with the nsy-1 gene from *Caenorhabditis elegans.* The GO-CAM models link out to the Gene Ontology website

Representation of pathways is supported using visualization widgets from Reactome (Gillespie et al. 2022) and from GO Causal Annotation Model (GO-CAM) curation (Thomas et al. 2019) which have been integrated into relevant gene pages on the Alliance web portal (Fig. 6). GO-CAMs are models of biological processes constructed by linking together individual GO annotations. The simplified models shown on Alliance gene pages are linked to model details at the Gene Ontology resource. The interactive Reactome pathways and reaction graphics on Alliance gene pages are linked to the Reactome database for additional information about the reactions and pathways.

### Phenotype annotations

If an organism has curated phenotype annotations, the annotations are displayed in a tabular format in the Phenotypes section of the gene detail page. The table includes columns for the phenotype term from the relevant phenotype ontology, annotation details, and the reference(s) for the annotation. Experimental conditions such as chemical, dietary, or physical interventions that contribute to or modify an observed phenotype are included in such annotation details are available. Although the display format is uniform for all organisms represented in the Alliance, the details displayed for phenotype annotations differ by organism. In mouse, for example, phenotype annotations are associated with genotypes and genetic backgrounds. In zebrafish, the phenotype annotations are associated with a fish. In *Drosophila*, phenotype annotations are associated with alleles.

### Disease associations and models

**Fig. 7:**
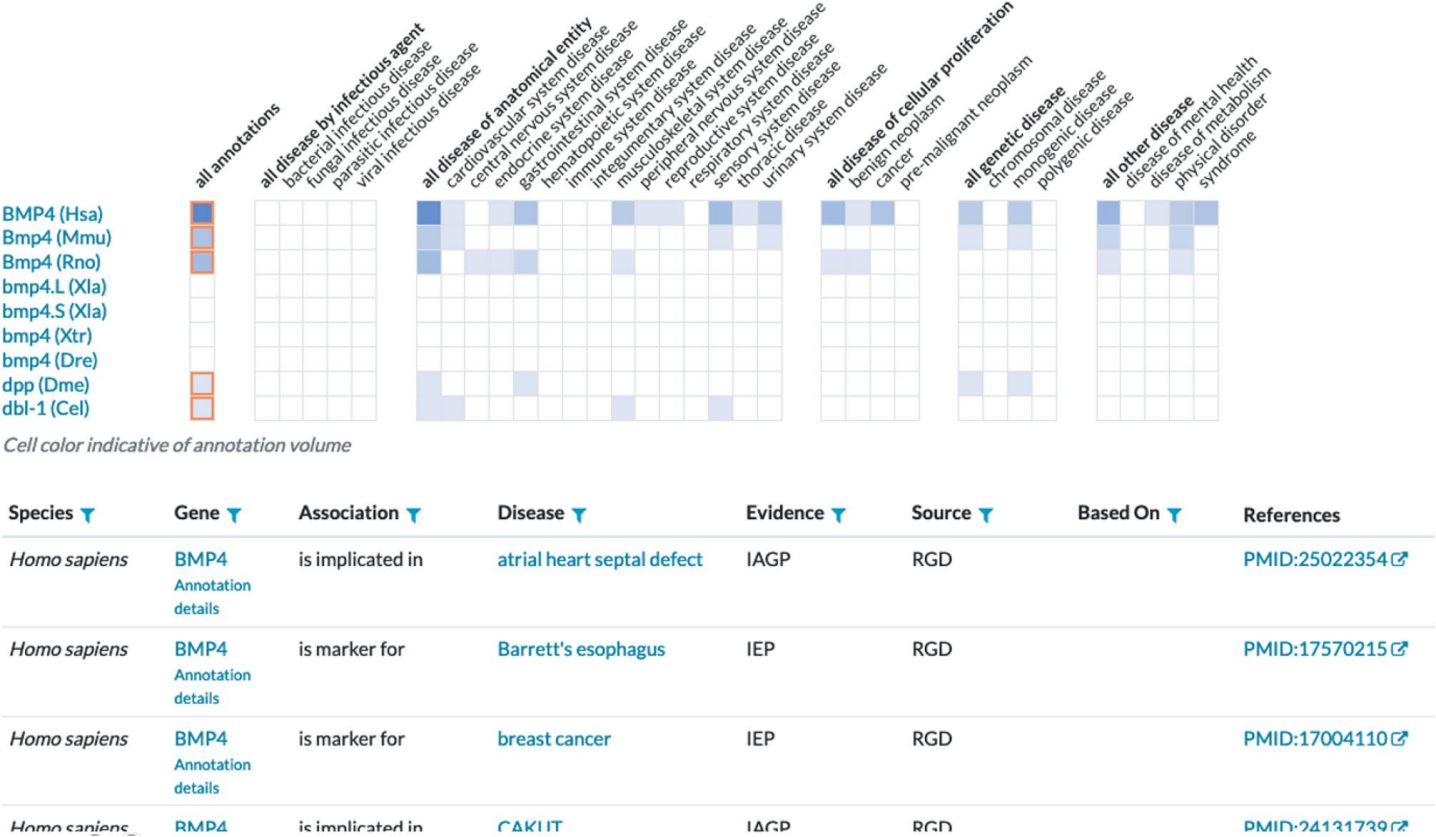
Partial screenshot of the comparative disease annotation summary on the *BMP4* gene detail page. The comparative annotation ribbon display is linked to a summary of disease associations for the *BMP4* gene. Disease annotations include the type of association, the disease ontology term, the evidence for the association, and the source(s) of evidence

Similar to the display for functional annotations, the summary of disease associations for a gene across available data across model organisms is displayed as an interactive ribbon (Fig. 7). Selecting a column in the ribbon generates a tabular summary of the annotations that includes annotation type, evidence, and source. Of the more than 350,000 disease model annotations available in version 5.4.0 of the Alliance website (Table 1), over 60,000 are from experimentally derived models. More than 28,800 annotations represent either biomarkers of disease or disease associations based on orthology to a human gene.

Disease Models are specific strains, genotypes, animals, etc. that support investigation into the genetic and genomic basis of phenotypes and disease. Models defined as genotypes that are associated with specific observable phenotypes and/or that have characteristics that reflect biological properties of specific human diseases or syndromes. As the harmonization for the concept of a model across different model systems is still being discussed, the details for model genotypes displayed on Alliance gene detail page are available as links back to the relevant Knowledge Center.

### Alleles, variants, and transgenes

Alleles and sequence variants for a gene are provided in a table format. Variants are defined as sequence differences at a single position or in contiguous nucleotides relative to a reference sequence. Variants are expressed in standard Human Genome Variation Society (den Dunnen et al. 2016) format and annotated with the variant type (e.g., SNP), variant identifier, and molecular consequence annotations generated by the Variant Effect Predictor (VEP) tool (McLaren et al. 2016). Alleles are defined as alternative forms of a gene and may be associated one or more sequence variants. Alleles are displayed with official nomenclature and synonyms and are linked to detail pages at the Alliance that summarize phenotype and disease associations when relevant.

The details provided for transgenic alleles varies by model organism but may include the symbol, the transgene construct, expressed components, knock-down targets, and regulatory regions. Transgenic alleles are linked to Alliance detail pages that provide transgene construct details and summaries of any annotated phenotype and/or disease associations.

### Sequence feature viewer

Every gene detail page includes a graphical summary of transcripts annotated to the gene. When relevant, the location of sequence variants associated with alleles of the gene are also displayed. Population level variants (polymorphisms) determined by high-throughput sequencing and/or large-scale genotyping technologies are not displayed in the feature viewer because of the volume and density of these data. High throughput variants and additional genome features can be viewed using the Alliance JBrowse instance (Buels et al. 2016). A link to JBrowse is provided under the sequence feature viewer widget.

### Gene expression

Expression data displayed using the standard Alliance interactive ribbon display. The data reflect developmental and cellular/anatomical expression of genes in wild-type backgrounds. As with the other comparative annotation ribbons on the gene detail pages, the shading of the cells is indicative of the number of annotations supporting the expression information, not levels of transcription or translation. Cells with red slashes indicate that a particular anatomical structure is not biologically relevant for the organism.

### Molecular and genetic interactions

Information about molecular and genetic interactions are available for genes from humans and all seven model organisms in the Alliance. The interaction data include curated information provided by two Alliance Knowledge Centers (WormBase and FlyBase) and two external interaction data resources: BioGrid (Oughtred et al. 2021) and IMex (Porras et al. 2022). Currently the interaction data are presented as a table but future plans for Alliance Central include the implementation of a graphical display for these data.

## Relating model organisms to human disease

Human disease annotations in the Alliance use standardized terms from the Disease Ontology (Schriml et al. 2022). As of version 5.4.0 of the Alliance database, over 11,200 DO terms are associated with model organism and/or human annotations (Table 1). Using the Alliance web portal, researchers can access disease annotations in one of two ways: keyword searching or via the interactive annotation ribbon graphic provided on gene detail pages.

**Fig. 8:**
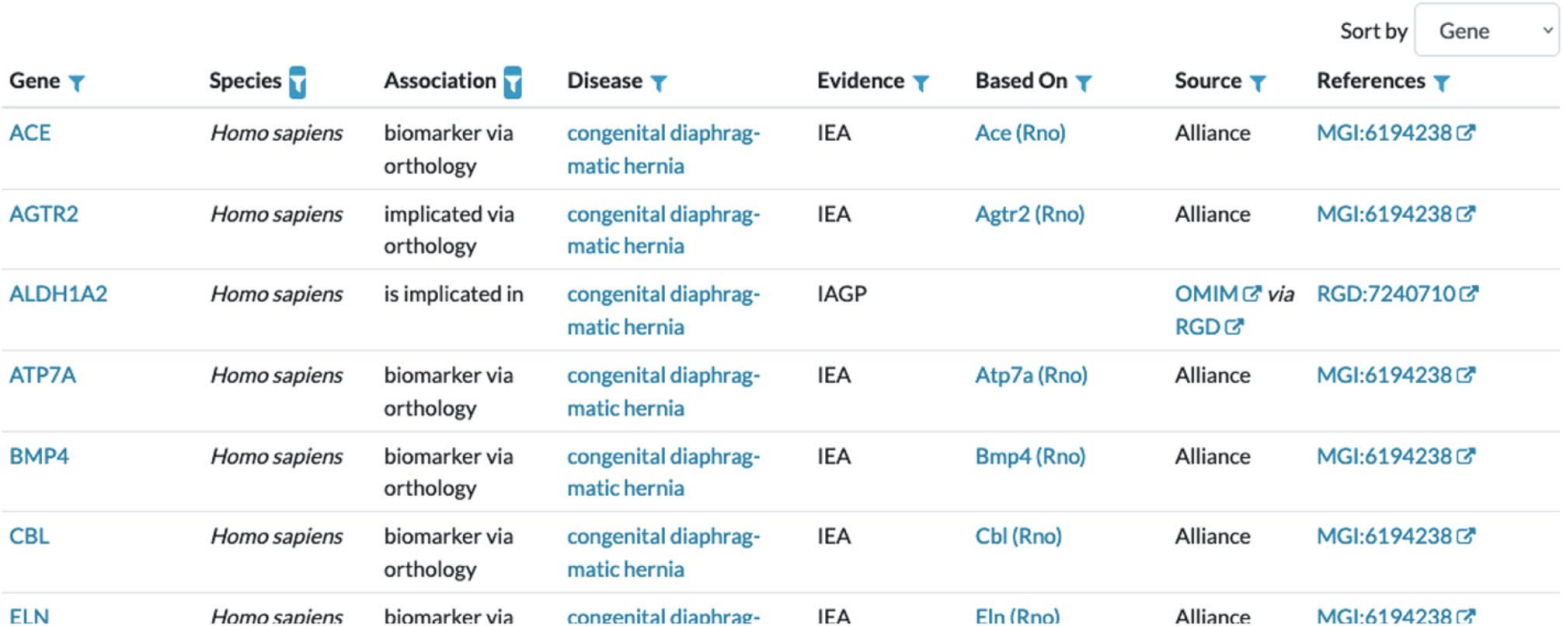
Partial screenshot of the results for a search of the disease term “congenital diaphragmatic hernia”. The results include genes associated with the disease, type of association and evidence, and the source(s) for the evidence

To facilitate keyword searches for a disease of interest, an autocomplete function dynamically generates a list of potential matching terms as the user types a term into the search form. Disease detail pages include a summary header containing the disease term definition from DO with cross references to other terminologies and ontologies. The multi-organism annotations available on disease detail pages include associated genes, alleles, and disease models. For associated genes, the nature of the association, the type of evidence for the association and the source(s) used to support the gene association are provided in an interactive table (Fig. 8). The types of disease gene associations listed include genes *implicated* as causal for a disease and those that are associated as *biomarkers* for a disease. Users can filter the rows displayed in the associated genes table by any of the column headers and can customize the sorting order by disease, gene, or species. Similar information and filter and sort functionality are provided for the associated alleles and associated models tables. Each of the tables shown on the disease detail pages can be downloaded as a tab-separated file.

## Extending the platform

One of the overarching goals of Alliance Central is to establish a knowledgebase platform capable of supporting model organism communities beyond the founding members of the Alliance consortium. A number of the software components developed by the Alliance have been adopted by external database resources, including the short gene descriptions, the Sequence Feature Viewer, and the annotation ribbon display. To demonstrate the extensibility of the platform to other model systems, the Alliance family of model organism databases recently was extended to include Xenbase, the model organism database for *Xenopus* sp.

Xenopus is a tetrapod model organism that occupies a key evolutionary position between the mammalian models and zebrafish already represented in the Alliance. Two species of Xenopus are now represented in the Alliance: The African clawed frog (*X. laevis*) and the Western clawed frog (*X. tropicalis*). Both *Xenopus* species are widely studied as models for developmental and cell biology. The African clawed frog, *X. laevis* (abbreviated Xla in the Alliance) is an allotetraploid (2*n* = 36) of hybrid origin. The resulting *X. laevis* genome has a set of ‘long’ and ‘short’ chromosomes and gene symbols are therefore appended with ‘.L’ or ‘.S’ denoting on which chromosome pair they reside. The second *Xenopus* species, the Western clawed frog *X. tropicalis* (Xtr), is a conventional diploid (2*n* = 20), and is increasingly used in modeling of human disease.

A key step in the integration of Xenopus into the Alliance was the modification of the representation of orthologs in the Alliance. Orthology assertions for *X. tropicalis* were generated from DIOPT. Orthology assertions for *X. laevis* were provided by Xenbase curators and are displayed as coming from the source, “Xenbase” on the orthology summary table. Data for both of the *Xenopus* species are available on gene detail pages, including feature gene descriptions, relationships to orthologs in other model organisms, disease associations for frog genes, gene expression, and a Sequence Feature Viewer.

## User support and community engagement

User support and engagement for the Alliance features a Help Desk, tutorials, an active social media presence, and an on-line discussion forum. Through the on-line forum researchers can share announcements about upcoming meetings and job postings and initiate dialog about organism-specific reagents and methods on the forum. From the Help menu on the Alliance home page researchers will find an extensive FAQ, glossary of terms, video tutorials, and on-line documentation describing how to access and use Alliance resources.

The Alliance offers workshops comprised lectures, demos, and interactive tutorials on a regular basis. Workshops are customized to the research interests and needs of the audience. To inquire about hosting an Alliance workshop (virtually or in person), email help@alliancegenome.org.

Access to the primary community engagement sites for the Alliance of Genome Resources consortium are as follows:

- Email access: help@alliancegenome.org
- Discussion forum: https://community.alliancegenome.org/
- Facebook: https://www.facebook.com/AllianceOfGenomeResources
- Twitter: https://twitter.com/alliancegenome
- YouTube: https://www.youtube.com/@allianceofgenomeresources9696/featured
- GitHub: https://github.com/alliance-genome

## Citing the alliance

For a general citation of the Alliance, researchers can cite this manuscript. For citing specific data or annotations, the recommended citation format is as follows:

[Type of] data for this paper were retrieved from the Alliance of Genome Resources, URL: https://www.alliancegenome.org; [date the data were retrieved and the release version of the resource].

The release version of the resource is found in the header of every web page (currently, 5.4.0).

## Summary and future directions

Prior to the formation of the Alliance of Genome Resources consortium and the Alliance Central knowledge commons platform, researchers seeking to compare biological and functional annotations across different model organisms were typically faced with the daunting task of navigating multiple web sites, each with its own unique style for user interfaces and APIs for programmatic data access. The Alliance of Genome Resources is transforming comparative genomics through the implementation of uniform display of and access to harmonized genetic and genomic data across diverse model organisms and human. Alliance resources allow researchers to easily find, access, compare, and analyze data across multiple species. The modular nature of the Alliance Central platform is designed specifically to allow extension of the resource to other model organisms which will benefit model organism research communities that lack centralized informatics resources by providing cost-effective infrastructure and data management practices that conform to FAIR principles (Wilkinson et al. 2016).

Future directions for the Alliance include the incorporation of additional intensively-studied model organisms into the platform, the continued harmonization of biological concepts, and refinement and expansion of novel interfaces and analysis tools in support of comparative biology and genomics. A major initiative currently underway within Alliance Central is the implementation of a centralized literature curation system that uses machine learning and artificial intelligence methods to (1) identify published manuscripts with data relevant to the mission of the Alliance and (2) map concepts and entities described in scientific publications to standard nomenclatures and ontology terms. This initiative builds on a large body of prior work among Alliance members to improve the efficiency and scalability of expert curation of knowledge published in the scientific literature (Hirschman et al. 2010; Karamanis et al. 2008; Liu et al. 2015; Muller et al. 2018; Ringwald et al. 2022).

## Data Availability

The annotations available from the Alliance of Genome Resources web portal (https://alliancegenome.org) are distributed under a CC BY 4.0 license.
